# Associative role of *HLA-DRB1* as a protective factor for susceptibility and progression of Parkinson’s disease: a Chinese cross-sectional and longitudinal study

**DOI:** 10.3389/fnagi.2024.1361492

**Published:** 2024-02-22

**Authors:** Raoli He, Yuqi Zeng, Chaodong Wang, Lina Chen, Guoen Cai, Ying Chen, Yingqing Wang, Qinyong Ye, Xiaochun Chen

**Affiliations:** ^1^Department of Neurology, Fujian Medical University Union Hospital, Fuzhou, China; ^2^Fujian Key Laboratory of Molecular Neurology and Institute of Neuroscience, Fujian Medical University, Fuzhou, China; ^3^Institute of Clinical Neurology, Fujian Medical University, Fuzhou, China; ^4^Department of Neurology, National Clinical Research Center for Geriatric Diseases, Xuanwu Hospital of Capital Medical University, Beijing, China

**Keywords:** Parkinson’s disease, single nucleotide polymorphism, *HLA-DRB1*, disease susceptibility, disease progression

## Abstract

**Background:**

Previous genome-wide association studies investigating the relationship between the *HLA-DRB1* and the risk of Parkinson’s disease (PD) have shown limited racial diversity and have not explored clinical heterogeneity extensively.

**Methods:**

The study consisted of three parts: a case–control study, a cross-sectional study, and a longitudinal cohort study. The case–control study included 477 PD patients and 477 healthy controls to explore the relationship between rs660895 and PD susceptibility. The cross-sectional study utilized baseline data from 429 PD patients to examine the correlation between rs660895 and PD features. The longitudinal study included 388 PD patients who completed a 3-year follow-up to investigate the effects of rs660895 on PD progression.

**Results:**

In the case–control study, *HLA-DRB1* rs660895-G allele was associated with a decreased risk of PD in allele model (adjusted OR=0.72, *p* = 0.003) and dominant model (AG + GG vs. AA: adjusted OR = 0.67, *p* = 0.003). In the cross-sectional analysis, there was no association between rs660895 and the onset age, motor phenotype, or initial motor symptoms. In the longitudinal analysis, PD patients with the G allele exhibited a slower progression of motor symptoms (MDS-UPDRS-III total score: *β* = −5.42, *p* < 0.001, interaction *p*_time × genotype_ < 0.001) and non-motor symptoms (NMSS score: *β* = −4.78, *p* = 0.030, interaction *p*_time × genotype_ < 0.001).

**Conclusion:**

Our findings support *HLA-DRB1* rs660895-G allele is a protective genetic factor for PD risk in Chinese population. Furthermore, we also provide new evidence for the protective effect of rs660895-G allele in PD progression.

## 1 Introduction

Parkinson’s disease (PD) is a multifactorial neurodegenerative disease characterized by complex pathogenesis. The pathophysiology of PD involves dopaminergic neuron death and α-synuclein accumulation (Lewy bodies) (Bloem et al., 2021). The study conducted by von Bernhardi et al. has revealed that enhanced microglial activation along with neuroinflammatory processes in the brain contributes to the progression of PD (von Bernhardi et al., 2015). Abnormalities in the peripheral immune system may drive onset and progression of PD by facilitating the immune cells infiltration and neuroinflammation in the central nervous system (Tansey and Romero-Ramos, 2019; Yan et al., 2021). Moreover, previous genome-wide association studies (GWAS) have identified numerous potential genetic loci associated with immune-inflammatory mechanisms, which may be strongly linked to susceptibility to PD (Satake et al., 2009; Simón-Sánchez et al., 2009; Nalls et al., 2019). This series of evidence from genetics and immunology further supports the involvement of immune-inflammatory mechanisms in PD (Simón-Sánchez et al., 2009; Herrero et al., 2015).

Human Leukocyte Antigen (*HLA*) gene, located on the major histocompatibility complex (MHC) on chromosome 6, encodes a group of proteins that bind peptides from foreign or self-antigens, such as α-synuclein. This binding allows T cells to recognize and subsequently coordinate the immune response. Previous genetic studies have shown that several single nucleotide polymorphisms (SNPs) in HLA region were associated with PD risk (Hamza et al., 2010; Nalls et al., 2011; Ahmed et al., 2012; Wissemann et al., 2013; Nalls et al., 2014). Particularly, the latest researches highlighted the strong correlation between PD and HLA-DR and HLA-DQ alleles within the HLA class II region (Naito et al., 2021). These specific HLAs may cause peptide-specific T cell responses, thereby involved in the pathogenesis of immune-inflammatory processes (Yu et al., 2021).

Due to geographical differences, there are extensive variations in *HLA* allele and haplotype frequencies among populations worldwide (Buhler and Sanchez-Mazas, 2011; Fernandez Vina et al., 2012). Previous studies have reported multiple loci in the *HLA-DR* region associated with PD. One of these loci is the SNP rs660895-G allele in the *HLA-DRB1* region, which has been confirmed by multiple studies in the European population (Ahmed et al., 2012; Wissemann et al., 2013; Chuang et al., 2017). Nevertheless, very few studies on the link between *HLA-DR* gene polymorphisms and PD have been conducted with Asian population (Foo et al., 2020), and the results are not consistent with previous large-scale European GWASs (Ahmed et al., 2012). Additionally, there is currently a scarcity of clinical studies investigating the relationship between the genetic heterogeneity of the *HLA-DRB1* gene and the clinical characteristics of PD, particularly in longitudinal studies. Given that, we focused on SNP rs660895 to verify the association of *HLA-DRB1* with risk of PD within the Han Chinese population. Furthermore, we aimed to delve deeper into the impact of this loci on the clinical characteristics and the trajectory of disease progression among PD patients.

## 2 Methods

### 2.1 Study design and data extraction

This multicenter, retrospective observational study conducted in China comprised three phases:

- Case–control study (1st phase): this sub-study assessed the association between *HLA-DRB1* rs660895 polymorphism and PD risk in Chinese individuals.
- Cross-sectional study (2nd phase): this sub-study investigated the association between clinical characteristics (e.g., onset age, motor phenotypes, and initial motor symptoms) and *HLA-DRB1* rs660895 genotypes.
- Longitudinal cohort study (3rd phase): this component examined the associations of *HLA-DRB1* rs660895 with changes of both motor and non-motor symptom over 3 years.

This study conformed to the code of Ethics of the World Medical Association Declaration of Helsinki and was approved by the Ethics Committee of Fujian Medical University Union Hospital (approval number: 2023KY177) and the Ethics Committee of Xuanwu Hospital of Capital Medical University (approval number: 2020060). All enrolled participants furnished written informed consent, which encompassed written informed consent for genetic studies.

Data for this study were obtained from an existing prospective observational cohort study, including consecutive individuals with PD from the Movement Disorders Clinic at Fujian Medical University Union Hospital (Fuzhou, China) and the Department of Neurology of Xuanwu Hospital of Capital Medical University (Beijing, China) between February 2017 and July 2023. All patients were diagnosed with idiopathic PD by two movement disorder neurologists and met the International Parkinson and Movement Disorder Society (MDS) Clinical Diagnostic Criteria for the clinical diagnosis of PD (Postuma et al., 2015). To minimize the effects of other interference factors, subjects with active or chronic inflammatory, autoimmune diseases, malignancy, immunodeficiency and any other neurodegenerative disorders were excluded from the study.

**Figure 1:**
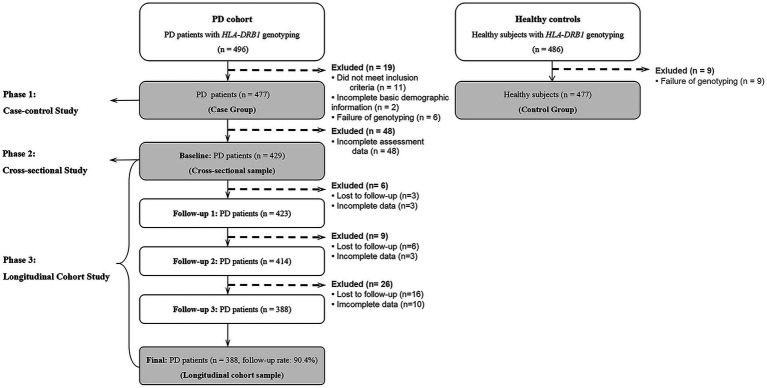
Study flow chart and analysis plan.

In the initial phase (the case–control study), alongside 477 PD patients whom were successfully typed for *HLA-DRB1* rs660895 and met the inclusion criteria, we also included 477 healthy individuals. These controls, genotyped for SNP rs660895 and devoid of any family history of PD, were randomly selected from 20 community units in Beijing and 5 in Fuzhou between April 2019 and September 2021. The next phase, a cross-sectional cohort study, focused on 429 of the 477 PD patients who had thorough baseline assessment data. These patients, who had complete baseline records and were tracked over a three-year span with annual clinical check-ups, progressed to the longitudinal cohort study. When considering consistent data availability throughout the follow-up, 388 (representing 90.4%) of the initial 429 PD patients were engaged in this longitudinal phase. All research subjects were of Han nationality. A comprehensive overview of the study’s methodology and patient inclusion is provided in Figure 1.

### 2.2 Clinical assessment

For each PD patient, a comprehensive clinical evaluation was conducted at baseline and during each annual follow-up visit over a period of 3 years. The data collected included medical history, medication usage, and family history. All participants underwent general medical and neurological examinations. Patients with age at onset ≤ 50 were classified as early-onset PD (EOPD), and age at onset ≥50 years as late-onset PD (LOPD) (Schrag and Schott, 2006). The levodopa equivalent daily dose (LEDD) was calculated based on published recommendations (Tomlinson et al., 2010). The Movement Disorders Society Unified Parkinson’s Disease Rating Scale (MDS-UPDRS) was used to assess Parkinson’s disease-related signs and symptoms (Goetz et al., 2008). The stage of the disease was categorized according to the modified Hoehn and Yahr stage (H&Y) (Goetz et al., 2004). The off-state Parkinsonian motor symptom progression was evaluated using Part 3 of the MDS-UPDRS. Motor phenotypes were determined based on the MDS-UPDRS score, classifying patients into tremor-dominant phenotype (TD), postural instability gait disorder phenotype (PIGD), or indeterminate phenotype. The ratio of tremor score to PIGD score was used to define patients with TD (ratio ≥ 1.15), patients with indeterminate phenotype (0.9 < ratio < 1.15), and patients with PIGD (ratio ≤ 0.9) (Stebbins et al., 2013). Additionally, cardinal motor features including tremor (items 20 and 21), rigidity (item 22), bradykinesia (items 23–26 and 31), and postural and gait abnormality (items 27–30) were calculated using the MDS-UPDRS for a more detailed assessment (Eisinger et al., 2020). The severity of overall non-motor dysfunction was assessed using the Non-motor Symptoms Scale (NMSS) (Chaudhuri et al., 2007). Global cognitive function was evaluated using the Montreal Cognitive Assessment (MoCA) (Nasreddine et al., 2005), and rapid eye movement sleep behavior disorder (RBD) was screened using the Rapid Eye Movement Sleep Behavior Disorder Screening Questionnaire (RBDSQ) (Stiasny-Kolster et al., 2007). Olfactory impairment was measured using a 12-item Sniffin’ Sticks test (SS-12) (Çomoğlu et al., 2015).

### 2.3 DNA extraction and genotyping

Genomic DNA was extracted from peripheral venous blood samples of all subjects using the Wizard Genomic DNA purification Kit (Promega, USA). Genotyping of the target SNP was performed using a matrix-assisted laser desorption/ionization time-of-flight (MALDI-TOF) mass spectrometer on the MassARRAY^Ⓡ^ Analyzer 4 platform (Sequenom Inc., San Diego, CA, USA). The target SNP call rate was 98.9%. Individuals with discordant gender information were removed. The genotype frequency distribution in the control group was consistent with Hardy–Weinberg Equilibrium (HWE) (*p* = 0.152, *p* > 0.05) (Supplementary Table S1). SNP genotyping and data analysis were conducted by CapitalBio Technology Inc., Beijing, China.

### 2.4 Statistical methods

The Fisher exact test was utilized to test for Hardy–Weinberg equilibrium (HWE). For the case–control analysis of this study, allelic and genotypic frequencies were analyzed using logistic regression analysis to compare PD patients and controls, with adjustments made for age and gender. Comparison of baseline cross-sectional parameters was performed by Student’s t test, the Mann–Whitney rank sum test, or the Chi-square test, as appropriate.

For the longitudinal cohort analysis, generalized linear mixed models (GLMMs) were utilized to evaluate the association between the SNP and changes in clinical characteristics over time. The fixed effects included genotype, time, and the interaction between genotype and time. Subjects were included as random effects. The models were presented as unadjusted model, minimally adjusted model (adjusted for age and gender), and fully adjusted model (adjusted for age, gender, onset age, disease course, and LEDDs). Furthermore, GLMMs with the variance components structure (including the time × genotype interaction term) were used to test the genotype effect on the trajectory of disease progression. *p* values less than 0.05 were considered statistically significant.

Statistical analysis was performed using IBM SPSS Statistics 24.0 (SPSS, Inc., Chicago, IL, USA), and figures were created using GraphPad Prism 7.0 (GraphPad Software, San Diego, CA, USA).

## 3 Results

### 3.1 Association of HLA-DRB1 rs660895 and PD risk in the case–control study

In this study, 477 PD patients (male: 235, female: 242; mean age: 64.1 ± 9.6 years) and 477 healthy controls (male: 207, female: 270, mean age: 64.0 ± 5.7 years) were included. The demographic and clinical characteristics of the study participants in the case–control study are presented in Table 1. The two groups in the case–control study were similar in terms of age at diagnosis, gender, and education level.

**Table 1:** Demographic and clinical characteristics of participants in the case–control study.

	PD (*n* = 477)	Control (*n* = 477)	*p*-value
Gender, male, *n* (%)	235 (49.3)	207 (43.4)	0.069
Age, years, mean ± SD	64.1 ± 9.6	64.0 ± 5.7	0.961
Education, years, median (IQR)	8.0 (6.0)	8.0 (5.0)	0.652
Age of disease onset, years, mean ± SD	58.1 ± 9.9	-	-
Duration of disease, years, median (IQR)	3.0 (5.0)	-	-

*n*, number; SD, standard deviation; IQR, interquartile range.

In our study, the frequency of the minor G-allele of rs660895 was 0.255 in healthy controls, closed to the 0.240 MAF in 1000 Genomes of East Asian populations (Auton et al., 2015). As shown in Table 2, the frequency of the G allele was significantly lower in individuals with PD compared to the control group (crude OR = 0.72, *p* = 0.003), indicating that the rs660895-G allele was strongly associated with a reduced risk of PD. This association remained statistically significant even after adjusting for age and gender (adjusted OR = 0.72, *p* = 0.003). Furthermore, we assessed the association between rs660895 and PD using three different genetic models (co-dominant, dominant and recessive). The results showed significant differences between cases and controls in both the co-dominant model (AG vs. AA: crude OR = 0.67, *p* = 0.005) and dominant model (AG + GG vs. AA: crude OR = 0.67, *p* = 0.003). After adjusting for age and gender, the association remained significant in the co-dominant model (AG vs. AA: adjusted OR = 0.68, *p* = 0.007) and the dominant model (AG vs. AA: adjusted OR = 0.67, *p* = 0.003). These findings suggest the *HLA-DRB1* rs660895-G allele is associated with the lower risk of PD in the Han Chinese population.

**Table 2:** Allele and genotype frequencies in *HLA-DRB1* rs660895 in cases and controls.

Genetic Model	*N* (Frequency)		OR (95%CI)	*p*-value	Adjusted for gender and age	
	PD *n* (%)	Control *n* (%)			Adjusted OR (95% CI)	*p_adj._* value
Allele						
A	765 (80.2)	711 (74.5)	Ref.	Ref.	Ref.	Ref.
G	189 (19.8)	243 (25.5)	0.72 (0.58–0.90)	**0.003** ^**^	0.72 (0.58–0.89)	**0.003** ^**^
Co-dominant						
AA	318 (66.7)	273 (57.2)	Ref.	Ref.	Ref.	Ref.
AG	129 (27.0)	165 (34.6)	0.67 (0.51–0. 89)	**0.005** ^**^	0.68 (0.51–0.90)	**0.007** ^**^
GG	30 (6.3)	39 (8.2)	0.66 (0.40–1.09)	0.106	0.64 (0.38–1.06)	0.080
Dominant						
AA	318 (66.7)	273 (57.2)	Ref.	Ref.	Ref.	Ref.
AG + GG	159 (33.3)	204 (42.8)	0.67 (0.51–0.87)	**0.003** ^**^	0.67 (0.51–0.87)	**0.003** ^**^
Recessive						
AA+AG	447 (93.7)	438 (91.8)	Ref.	Ref.	Ref.	Ref.
GG	30 (6.3)	39 (8.2)	0.75 (0.46–1.24)	0.262	0.72 (0.44–1.19)	0.203

*n*, number; OR, odds ratio; CI, confidence interval; Ref., reference. Boldface indicates statistical significance (^*^p < 0.05, ^**^p < 0.01, ^***^p < 0.001).

In addition, considering that epigenetic modifications are influenced by geographical traits, we conducted a comparative analysis of samples derived from Fuzhou and Beijing populations. The analysis revealed no significant differences in demographics and genotype composition between two groups (Supplementary Table S2). And after adjusting the model based on different centers, consistent protection against the risk of PD was observed with the rs660895-G allele (Supplementary Table S3 for detail).

### 3.2 Association of HLA-DRB1 rs660895 and clinical profiles of PD in the cross-sectional study

Under the dominant model of rs660895, the baseline cross-sectional study included 429 PD patients who were divided into two *HLA-DRB1* genotype groups: the G allele carriers (AG + GG) and the G allele non-carriers (AA). No significant difference in the demographic characteristics between groups. Additional analysis of PD motor characteristics revealed no significant association between rs660895-G allele carrier status and age of onset (*p* = 0.352), motor phenotype (*p* = 0.743), or initial motor symptoms (*p* = 0.293). The detailed data be shown in the Table 3.

**Table 3:** Demographic and clinical characteristics of PD patients under the dominant model for *HLA-DRB1* rs660895 in the cross-sectional cohort study (*n* = 429).

Characteristic	Groups		*p*-value
	G allele carrier	G allele non-carrier	
	(*n* = 169)	(*n* = 260)	
Demographics information			
Gender, male, *n* (%)^a^	112 (61.5)	148 (56.9)	0.343
Age, years, mean ± SD^b^	63.5 ± 9.7	63.0 ± 9.7	0.591
Age of disease onset, *n* (%)^a^			
EOPD	32 (18.9)	59 (22.7)	0.352
LOPD	137 (81.1)	142 (77.3)	
Duration of disease, years, median (IQR)^c^	3.0 (5.0)	3.0 (5.0)	0.905
Education, years, median (IQR)^c^	8.0 (6.0)	8.0 (6.0)	0.535
Modified H&Y Stages, *n* (%)^a^			
Early stage (stages 1.0–2.5)	148 (87.6)	224 (86.2)	0.672
Advanced stage (stages 3.0–5.0)	21 (12.4)	36 (13.8)	
LEDDs, mg, median (IQR)^c^	300.0 (275.0)	300.0 (262.5)	0.190
Motor features			
Motor Phenotype, *n* (%)^a^			
TD	46 (27.2)	74 (28.5)	0.743
PIGD	105 (62.1)	164 (63.0)	
Indeterminate	18 (10.7)	22 (8.5)	
Initial motor symptom, *n* (%)^a^			
Bradykinesia or rigidity	68 (40.2)	118 (45.4)	0.293
Tremor	101 (59.8)	260 (54.6)	
MDS-UPDRS-III score, median (IQR)^c^	20.0 (15.0)	20.0 (16.0)	0.971
Non-motor features			
NMSS score, median (IQR)^c^	27.0 (23.0)	23.0 (27.0)	0.431
MoCA score, median (IQR)^c^	23.0 (7.0)	22.5 (7.0)	0.769
RBDSQ score, median (IQR)^c^	1.0 (4.0)	1.0 (3.0)	0.964
SS-12 score, median (IQR)^c^	5.0 (4.0)	5.0 (3.0)	0.785

^a^Chi square was used; ^b^Student’s t-test was used; ^c^Mann–Whitney U test was used. *n*, number; EOPD, Early-onset Parkinson’s disease patients; LOPD, Late-onset Parkinson’s disease patients; LEDDs, L-Dopa equivalent daily dosages; TD, tremor dominant phenotype; PIGD, postural instability and gait difficulty phenotype; MDS-UPDRS-III, Part III of Movement Disorders Society Unified Parkinson’s Disease Rating Scale; NMSS, Non-motor Symptoms Scale; MoCA, Montreal Cognitive Assessment; RBDSQ, Rapid Eye Movement Sleep Behavior Disorder Screening Questionnaire; SS-12, 12-item Sniffin’ Sticks test; SD, standard deviation; IQR, interquartile range.

### 3.3 Association of HLA-DRB1 rs660895 and disease progression of PD in the longitudinal cohort study

We further explored the relationship between the rs660895-G allele and disease progression in patients with PD. Under the dominant model, the study included a total of 388 patients with complete data. This comprised of 152 G allele carriers (AG + GG) and 236 G allele non-carriers (AA). There were no significant differences between the two trajectories in demographic and clinical variables at baseline. The retention rate was also similar between G allele carriers (89.9%) and G allele non-carriers (90.8%) (*p* = 0.776). The participants lost to follow-up had similar baseline characteristics to those who completed the study. Supplementary Table S4 provides a summary of the demographic and clinical parameters of the longitudinal analyzed population at baseline in detail.

#### 3.3.1 Effect of *HLA-DRB1* rs660895 on the progression of motor symptoms in PD patients

**Figure 2:**
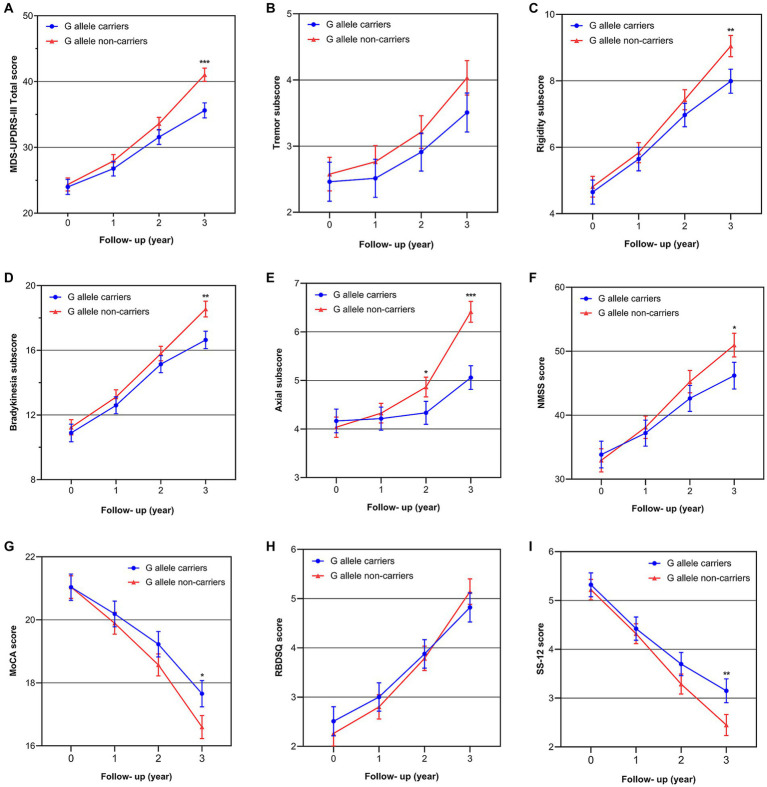
The best-fit trajectories of motor symptom scores and non-motor symptom scores over 3 years in PD patients according to the G allele of *HLA-DRB1* rs660895 (in the fully adjusted model). **(A)** MDS-UPDRS-III total score; **(B)** tremor subscore; **(C)** rigidity subscore; **(D)** bradykinesia subscore; **(E)** axial score; **(F)** NMSS score; **(G)** MoCA score; **(H)** RBD-SQ score; **(I)** SS-12 score.

The results of our study indicated that SNP rs660895-G allele showed a strong influence on the progression of motor symptoms over time. As shown in the three-year trajectories of motor symptoms progression (Figures 2A–E), patients with the G allele had lower MDS-UPDRS-III total score, rigidity subscore, bradykinesia subscore, and axial subscore at the 3rd year follow-up in the unadjusted model (MDS-UPDRS-III total score: *p* < 0.001; rigidity subscore: *p* = 0.006; bradykinesia subscore: *p* = 0.001; axial subscore: *p* < 0.001), except for tremor subscore (*p* = 0.090). These associations remained significant even in the minimally adjusted model and the fully adjusted model (Table 4). We further assessed the main effect of time, genotype, and the interaction of time and genotype, showing that the effect of genotype on overall motor symptoms, rigidity, bradykinesia, and axial impairment significantly varied by the time of assessment. Longitudinal analysis showed genotype effect with a significant genotype effect and genotype-by-time interaction on overall motor symptoms and axial impairment (MDS-UPDRS-III total scores: *p*_genotype_ = 0.032 and *p*_time×genotype_ < 0.001 in model 2, *p*_genotype_ = 0.047 and *p*_time×genotype_ < 0.001 in model 3; Axial subscore: *p*_genotype_ = 0.040 and *p*_time×genotype_ < 0.001 in model 1, *p*_genotype_ = 0.021 and *p*_time×genotype_ < 0.001 in model 2, *p*_genotype_ = 0.042 and *p*_time×genotype_ < 0.001 in model 3). And the genotype-by-time interaction for rigidity and bradykinesia was significant, although there was no significant genotype effect (Rigidity subscores: *p*_time×genotype_ = 0.011 in model 1, *p*_time×genotype_ = 0.011 in model 2, *p*_time×genotype_ = 0.023 in model 3; Bradykinesia subscore: *p*_time×genotype_ < 0.001 in model 1, *p*_time×genotype_ < 0.001 in model 2, *p*_time×genotype_ = 0.002 in model 3). Detailed information about the fixed-effects statistical analyses on motor symptoms is in Supplementary Table S5.

**Table 4:** Changes in motor symptoms and non-motor symptoms scores according to *HLA-DRB1* rs660895 genotypes under the dominant model.

	Model 1			Model 2			Model 3		
	*β*	SE	*p*-value	*β*	SE	*p*-value	*β*	SE	*p*-value
Baseline									
MDS-UPDRS-III total score	−0.37	1.30	0.779	−0.59	1.28	0.644	−0.37	1.21	0.762
Tremor subscore	−0.13	0.33	0.686	−0.14	0.33	0.670	−0.12	0.32	0.717
Rigidity subscore	−0.14	0.40	0.721	−0.21	0.39	0.603	−0.16	0.39	0.674
Bradykinesia subscore	−0.36	0.60	0.541	−0.43	0.59	0.463	−0.35	0.57	0.544
Axial subscore	0.11	0.29	0.713	0.06	0.28	0.842	0.13	0.26	0.624
NMSS score	0.93	2.13	0.664	0.64	2.36	0.785	0.90	2.19	0.679
MoCA score	0.06	0.50	0.906	0.07	0.44	0.884	0.00	0.45	0.995
RBDSQ score	0.25	0.32	0.432	0.21	0.32	0.520	0.25	0.31	0.429
SS-12 score	0.07	0.24	0.787	0.12	0.26	0.651	0.10	0.26	0.696
Follow-up 1 (1 year)									
MDS-UPDRS-III total score	−1.21	1.30	0.353	−1.44	1.28	0.262	−1.17	1.21	0.334
Tremor subscore	−0.27	0.33	0.409	−0.28	0.33	0.397	−0.25	0.32	0.428
Rigidity subscore	−0.18	0.40	0.651	−0.24	0.39	0.538	−0.19	0.39	0.620
Bradykinesia subscore	−0.57	0.60	0.339	−0.64	0.59	0.279	−0.51	0.57	0.373
Axial subscore	−0.15	0.29	0.594	−0.20	0.28	0.472	−0.11	0.26	0.665
NMSS score	−0.91	2.13	0.670	−1.19	2.36	0.613	−0.92	2.19	0.675
MoCA score	0.38	0.50	0.450	0.38	0.44	0.386	0.30	0.45	0.505
RBDSQ score	0.20	0.32	0.538	0.15	0.32	0.638	0.20	0.31	0.521
SS-12 score	0.07	0.24	0.761	0.13	0.26	0.628	0.10	0.26	0.688
Follow-up 2 (2 years)									
MDS-UPDRS-III total score	−2.14	1.30	0.101	−2.36	1.28	0.066	−2.04	1.21	0.093
Tremor subscore	−0.32	0.33	0.320	−0.33	0.33	0.310	−0.30	0.32	0.337
Rigidity subscore	−0.46	0.40	0.247	−0.52	0.39	0.185	−0.46	0.39	0.234
Bradykinesia subscore	−0.69	0.60	0.247	−0.76	0.59	0.198	−0.66	0.58	0.254
Axial subscore	−0.59	0.29	**0.039** ^*^	−0.64	0.28	**0.023** ^*^	−0.53	0.26	**0.043** ^*^
NMSS score	−2.69	2.13	0.206	−2.98	2.36	0.207	−2.61	2.19	0.232
MoCA score	0.76	0.50	0.127	0.77	0.44	0.082	0.66	0.45	0.141
RBDSQ score	0.08	0.32	0.812	0.03	0.32	0.928	0.09	0.31	0.773
SS-12 score	0.39	0.24	0.112	0.44	0.26	0.090	0.41	0.26	0.113
Follow-up 3 (3 years)									
MDS-UPDRS-III total score	−5.71	1.30	**<0.001** ^***^	−5.94	1.28	**<0.001** ^***^	−5.42	1.22	**<0.001** ^***^
Tremor subscore	−0.55	0.33	0.090	−0.56	0.33	0.086	−0.52	0.32	0.099
Rigidity subscore	−1.10	0.40	**0.006** ^**^	−1.17	0.39	**0.003** ^**^	−1.06	0.39	**0.007** ^**^
Bradykinesia subscore	−2.04	0.60	**0.001** ^**^	−2.11	0.59	**<0.001** ^***^	−1.91	0.58	**0.001** ^**^
Axial subscore	−1.48	0.29	**<0.001** ^***^	−1.53	0.28	**<0.001** ^***^	−1.36	0.26	**<0.001** ^***^
NMSS score	−5.23	2.13	**0.014** ^*^	−5.52	2.36	**0.019** ^*^	−4.78	2.20	**0.030** ^*^
MoCA score	1.25	0.50	**0.012** ^*^	1.26	0.44	**0.005** ^**^	1.06	0.45	**0.018** ^*^
RBDSQ score	−0.36	0.32	0.260	−0.41	0.32	0.198	−0.32	0.31	0.308
SS-12 score	0.70	0.24	**0.004** ^**^	0.76	0.26	**0.004** ^**^	0.70	0.26	**0.007** ^**^

Model 1—unadjusted model; Model 2—minimally adjusted model, adjusted for age and gender; Model 3—fully adjusted model, adjusted for age, gender, onset age, disease course, and LEDDs; MDS-UPDRS-III, Part III of Movement Disorders Society Unified Parkinson’s Disease Rating Scale; NMSS, Non-motor Symptoms Scale; MoCA, Montreal Cognitive Assessment; RBDSQ, Rapid Eye Movement Sleep Behavior Disorder Screening Questionnaire; SS-12, 12-item Sniffin’ Sticks test. *β* represents changes in outcomes by carrying G alleles of rs660895. *p* value for comparison of the evolution over time (results of GLMMs). Boldface indicates statistical significance (^*^p < 0.05, ^**^p < 0.01, ^***^p < 0.001).

#### 3.3.2 Effect of HLA-DRB1 rs660895 on the progression of non-motor symptoms in PD patients

As shown in the three-year trajectories of over-all non-motor symptoms progression (Figure 2F), patients with the G allele had lower NMSS score at the 3rd year follow-up (*p* = 0.0140 in model 1; *p* = 0.019 in model 2; *p* = 0.030 in model 3) (Table 4). Longitudinal analysis showed that NMSS score increased with time in two groups (*p*_time_ < 0.001 in all models). No significant group effect (*p*_genotype_ > 0.05 in all models), while a significant genotype-by-time interaction effect was found (*p*_time×genotype_ < 0.001 in all models).

Given the aforementioned results demonstrating the obvious effect of the G allele on the progression of overall non-motor symptoms, subsequent longitudinal analysis was conducted to assess the impact of the G allele status on cognitive impairment, RBD, and olfactory impairment. Our findings revealed significant time effects and time-genotype interactions on the above three aspects, but no significant group effects. Additionally, we observed three-year trajectories of the MoCA and SS-12 scores, which showed that patients with the G allele had better MoCA score (*p* = 0.012 in model 1; *p* = 0.005 in model 2; *p* = 0.018 in model 3) and SS-12 score (*p* = 0.004 in model 1; *p* = 0.004 in model 2; *p* = 0.007 in model 3) (Figures 2G–I; Table 4). It is worth noting that, although there was an interaction effect of genotype and time on RBD, the differences in the RBD-SQ score between different genotype groups did not reach statistical significance at the 3-year follow-up (*p* > 0.05 in all models) (Figure 2H; Table 4). Detailed information about the fixed-effects statistical analyses on non-motor symptoms is in Supplementary Table S6.

## 4 Discussion

This study replicated and verified an inverse relationship between *HLA-DRB1* rs660895 and susceptibility to PD in Chinese Han population. Importantly, through subsequent gene-based analysis in a 3-year PD cohort, we discovered that the rs660895-G allele may has a protective effect on the longitudinal changes in both motor and non-motor symptoms of PD. This finding demonstrates the potential importance of *HLA* gene in the long-term progression of PD.

The highly polymorphic *HLA* gene plays a crucial role in acquired immunity. Several studies have explored the relationship between the *HLA* gene and PD in recent years (Supplementary Table S7). *HLA* gene, especially *HLA-DRB1*, widely regarded relevant to the susceptibility of PD in Caucasian population, although specifics loci differ (Hamza et al., 2010; Guo et al., 2011; Nalls et al., 2011; Ahmed et al., 2012; Wissemann et al., 2013; Nalls et al., 2014; Chuang et al., 2017; Hollenbach et al., 2019; Chang et al., 2020;Naito et al., 2021; Yu et al., 2021; Le Guen et al., 2023). The latest research once again confirmed that specific *HLA-DRB1* variants are associated with a reduced risk of PD and suggested that this protective effect is primarily caused by specific amino acid polymorphisms present in most HLA-DRB1*04 subtypes (Naito et al., 2021; Yu et al., 2021; Le Guen et al., 2023). In Asians, the association between *HLA* and PD in GWASs is much less. Although Naito et al. identified *HLA-DRB1* locus (rs504594) associated with HLA-DRB1*04 in East Asians (Naito et al., 2021), it is important to note that this finding was not replicated in two other PD-related GWAS in Asia (Foo et al., 2020; Pan et al., 2023), which did not identify any *HLA* alleles. Thus, in this study, we focused on the Chinese Han population and conducted a comprehensive analysis to investigate the relationship between PD and rs660896, which has a strong correlation with HLA-DRB1*04. Our findings suggest that the G allele of significantly decreases the risk of PD in different genetic inheritance models, including allele, co-dominant, and dominant models. Particularly in the dominant model, the risk of PD was found to be approximately 33–36% lower compared to the healthy control group. These results confirm the strong correlation between rs660895 and the risk of PD. While this finding appears to be inconsistent with those reported in previous studies in Asian (Foo et al., 2020; Naito et al., 2021), it is consistent with previous studies conducted in Caucasian populations (Ahmed et al., 2012; Chuang et al., 2017).

Moreover, PD is a disorder with a highly variable clinical phenotype. Understanding genetic variants that modify disease presentation and progression is crucial for gaining insights into disease mechanisms and potential therapeutic targets. Unfortunately, subsequent cross-sectional analysis grouped based on the dominant inheritance model has not shown that possessing the rs660896 G allele affects the onset age, motor phenotypes, and initial motor symptoms of PD. Meanwhile, certain studies have identified a temporal longitudinal connection between α-synuclein-specific T cell reactivity and PD (Lindestam Arlehamn et al., 2020). Thus, considering the positive effect of rs660895 on reducing the risk of PD in this study, we further extended our observations to evaluate whether the rs660895 also affects symptoms progression in our 3-year PD cohort. Notably, our longitudinal study found that *HLA-DRB1* rs660895 showed evidence of strong protection against the progression of PD. During the 3-year follow-up period, both G allele carriers and non-carriers generally exhibited a decline in terms of overall motor symptoms (including rigidity, bradykinesia, and axial impairment), overall non-motor symptoms, cognitive impairment, RBD, and olfactory impairment. Nevertheless, PD patients carrying the protective G allele exhibited an obviously slower rate of both motor and non-motor symptoms progression, particularly after the 2nd year of follow-up. This suggests that the beneficial effect of the G allele is continuing, and appears greater for a long-term impact on PD progression over time. This novel evidence highlights the previously unexplored correlation between PD progression and *HLA* genotypes.

Although the causal role of *HLA* molecules in the pathogenesis of PD is not fully understood, recent studies have shown that α-synuclein peptides can induce differential T cell reactivity associated with *HLA* alleles (Sulzer et al., 2017; Lindestam Arlehamn et al., 2020). Yu et al. further conducted trans-ethnic fine-mapping of the MHC region and discovered that HLA-DRB1*04 subtypes (*HLA-DRB1* alleles with His13) may have a protective role in the development of PD by reducing the binding affinity to α-synuclein epitopes (Yu et al., 2021). Thus, we hypothesize that the polymorphism of rs660895, which has been previously linked to a strong association with HLA-DRB1*04, could potentially impede T cell activation by altering the binding affinity between *HLA-DRB1* molecules and α-synuclein. However, further research is needed to explore the precise amino acid positions and the epitope presentation to α-synuclein peptides.

This study had several noteworthy strengths, limitations and uncertainties. One key strength of our study is that, despite having a smaller sample size compared to previous large-scale GWAS studies, we were able to observe the role of rs660895 in the progression of PD through a longitudinal cohort with an excellent follow-up rate and detailed clinical information. This is a rare opportunity to identify potential protective trends in *HLA-DRB1* with disease progression, providing additional clinical support toward the hypothesis that neuroinflammation mediates the progression of PD. Another unique feature of this study is that it analyzed a specific Asian population (i.e., ethnic Han Chinese), a previously under-studied group. Given the limited number of PD-related GWAS studies conducted on Asians, our research contributes to expanding the applicability of previous findings. Specifically, we investigate whether the *HLA-DRB1* rs660895 polymorphism has a protective effect on PD in Asian populations.

However, there are limitations to consider in the interpretation of our findings. First, our study only focused on a single SNP, rs660895, located in *HLA-DRB1* gene. Although we have discovered an association of rs660895 with the risk and progression of PD, it is still unknown whether we have identified the functional variant. Additionally, due to limitations in follow-up duration and patient compliance issues with genetic testing, we were unable to test other newly discovered potential *HLA*-related loci and impute and separate the HLA-DRB1*04:05 with other *DRB1* variants. It may have influenced our evaluation of the protective strength of rs660895 to some extent, as it is likely that rs660895 is only in linkage disequilibrium with the causal SNP. Hence, future studies should consider conducting a more comprehensive linkage analysis to address this issue. Second, despite many clinical variables, there were no adjustments for multiple comparisons in the longitudinal analyses. It is worth noting that the severity of non-motor symptoms (secondary indicators) is closely associated with motor symptoms (main indicators) (Santos-García et al., 2020). Thus, the results of our longitudinal exploratory study are hypothesis generating and focused on magnitudes of differences rather than statistical significance on disease progression. Additional high quality longitudinal studies are needed to further evaluate this protective effect.

In conclusion, this study comprehensively investigates the relationship between *HLA-DRB1* rs660895 and PD in the Han Chinese population. The findings clearly demonstrate that the rs660895-G allele is associated with a decreased risk of PD. Furthermore, the study provides additional evidence suggesting that this allele may contribute to slowing the progression of both motor and non-motor symptoms of PD. Despite some limitations, the findings of this study enhance our understanding of the genetic factors underlying PD in the Chinese population and offer a new gene target for therapeutic development.

## Data availability statement

The data analyzed in this study is subject to the following licenses/restrictions: the data presented in this study are available on request from the corresponding author. The data are not publicly available due to privacy or ethical restrictions. Requests to access these datasets should be directed to unionqyye8@fjmu.edu.cn.

## Ethics statement

The studies involving humans were approved by Ethics Committee of Fujian Medical University Union Hospital Ethics Committee of Xuanwu Hospital of Capital Medical University. The studies were conducted in accordance with the local legislation and institutional requirements. The participants provided their written informed consent to participate in this study.

## Author contributions

RH: Data curation, Formal analysis, Investigation, Writing – original draft. YZ: Investigation, Writing – original draft. CW: Data curation, Resources, Writing – review & editing. LC: Data curation, Investigation, Writing – review & editing. GC: Formal analysis, Methodology, Writing – review & editing. YC: Data curation, Writing – review & editing. YW: Data curation, Writing – review & editing. QY: Conceptualization, Funding acquisition, Supervision, Writing – review & editing, Resources. XC: Conceptualization, Project administration, Supervision, Writing – review & editing.
