# Supplementary material for: Associative role of HLA-DRB1 as a protective factor for susceptibility and progression of Parkinson’s disease: a Chinese cross-sectional and longitudinal study
Source: Front Aging Neurosci. 2024 Feb 22;16:1361492. doi: 10.3389/fnagi.2024.1361492 (PMC10995924; doi:10.3389/fnagi.2024.1361492)
Supplement: Supplementary file 1 [file Data_Sheet_1.DOCX]

Supplementary Material

# Supplementary Tables

**Table S1.** Hardy-Weinberg equilibrium of *HLA-DRB1* rs660895 between PD patients and controls

|  | **Genotype** | **Observed number** | **Predicted number** | **HWE** | |
| --- | --- | --- | --- | --- | --- |
|  |  |  |  | **χ^2^** | ***p* value** |
| **PD** | AA | 318 | 306.62 | 10.57 | 0.005 |
|  | AG | 129 | 151.56 |  |  |
|  | GG | 30 | 18.72 |  |  |
| **Control** | AA | 273 | 264.95 | 3.77 | 0.152 |
|  | AG | 165 | 181.10 |  |  |
|  | GG | 39 | 30.95 |  |  |

*HWE* Hardy–Weinberg equilibrium. *p* value >0.05 was considered equilibrium.

**Table S2. The comparison of the demographic characteristics of participant between different centers in the case-control study**

|  | **PD (n = 477)** | | |  | **Control (n = 477)** | | |
| --- | --- | --- | --- | --- | --- | --- | --- |
|  | **Fujian-derived**  **(n = 375)** | **Beijing-derived**  **(n =102)** | ***p***  **value** |  | **Fujian-derived**  **(n= 339)** | **Beijing-derived**  **(n =138)** | ***p***  **value** |
| **Gender, male, n (%)** ^a^ | 182 (48.5) | 53 (52.0) | 0.539 |  | 156 (46.0) | 51 (37.1) | 0.070 |
| **Age, years, mean ± SD** ^b^ | 64.3 ± 9.6 | 62.8 ± 9.6 | 0.158 |  | 63.6 ± 6.3 | 64.2±4.5 | 0.117 |
| **Genotype, n (%)** ^a^ |  |  |  |  |  |  |  |
| **AA** | 252 (67.2) | 66 (64.7) | 0.889 |  | 195 (57.5) | 78 (56.5) | 0.959 |
| **AG** | 100 (26.7) | 29 (28.4) |  |  | 117 (34.5) | 48 (37.8) |  |
| **GG** | 23 (6.1) | 7 (6.9) |  |  | 27 (8.0) | 12 (8.7) |  |

^a^ Chi square was used; ^b^ Student's *t*-test was used.

**Table S3.** Allele and genotype frequencies in *HLA-DRB1* rs660895 in cases and controls (adjusted for gender, age, and center-derived)

| **Genetic Model** | **Adjusted Model** | | |
| --- | --- | --- | --- |
|  | **OR** | **95% CI** | ***p_adj._* value** |
| **Allele** | | | |
| A | Ref. | Ref. | Ref. |
| G | 0.72 | 0.58~0.90 | **0.003^**^** |
| **Co-dominant** | | | |
| AA | Ref. | Ref. | Ref. |
| AG | 0.68 | 0.51~0.90 | **0.007^**^** |
| GG | 0.64 | 0.39~1.07 | 0.087 |
| **Dominant** | | | |
| AA | Ref. | Ref. | Ref. |
| AG+GG | 0.67 | 0.52~0.87 | **0.003^**^** |
| **Recessive** | | | |
| AA+AG | Ref. | Ref. | Ref. |
| GG | 0.73 | 0.44~1.20 | **0.215** |

Abbreviations: n, number; OR, odds ratio; CI, confidence interval; Ref., reference.

Boldface indicates statistical significance (^*^ *p* < 0.05, ^**^ *p* < 0.01, ^***^ *p* <0.001).

**Table S****4.** Demographic and clinical characteristics of PD patients under the dominant model for *HLA-DRB1* rs660895 at baseline in the longitudinal cohort study (final analysis: n=388)

|  | **G allele carriers** | | |  | **G allele non-carriers** | | |  | ***p* value** ^c^ |
| --- | --- | --- | --- | --- | --- | --- | --- | --- | --- |
|  | **Follow-up** | **Lost to Follow-up** | ***p*  value** ^a^ |  | **Follow-up** | **Lost to Follow-up** | ***p*  value** ^b^ |  |  |
|  | **n = 152** | **n = 17** |  |  | **n=236** | **n = 24** |  |  |  |
| **Gender, male, n (%)** ^d^ | 93 (61.2) | 11 (64.7) | 0.777 |  | 134 (56.8) | 14 (58.3) | 0.884 |  | 0.39 |
| **Age, years, mean ± SD** ^e^ | 63.5 ± 9.6 | 63.6 ± 9.2 | 0.954 |  | 62.8 ± 9.5 | 64.3 ±12.0 | 0.492 |  | 0.525 |
| **Age of disease onset, mean ± SD** ^e^ | 59.1 ± 10.2 | 59.8 ± 8.3 | 0.765 |  | 58.3 ±10.2 | 60.1 ± 13.1 | 0.428 |  | 0.269 |
| **Duration of disease, years, median (IQR)** ^f^ | 3.0 (5.0) | 3.0 (4.0) | 0.498 |  | 3.0 (5.75) | 3.0 (2.75) | 0.599 |  | 0.935 |
| **Education, years, median (IQR)** ^f^ | 8.0 (6.0) | 8.0 (8.5) | 0.198 |  | 8.0 (6.0) | 6.6 (6.8) | 0.158 |  | 0.491 |
| **Modified H&Y Stages, n (%)** ^d^ |  |  |  |  |  |  |  |  |  |
| Early stage (stages 1.0- 2.5) | 135 (88.8) | 13 (76.5) | 0.282 |  | 202 (85.6) | 22 (91.7) | 0.61 |  | 0.359 |
| Advanced stage (stages 3.0- 5.0) | 17 (11.2) | 4 (23.5) |  |  | 34 (14.4) | 2 (8.3) |  |  |  |
| **LEDDs at baseline, mg, median (IQR)** ^f^ | 300.0 (262.5) | 300.0 (225.0) | 0.468 |  | 300.0 (259.4) | 287.5 (377.4) | 0.626 |  | 0.232 |
| **Motor symptoms** |  |  |  |  |  |  |  |  |  |
| - MDS-UPDRS-III total score, median (IQR) ^f^ | 20.0 (15.0) | - | - |  | 20.0 (17.0) | - | - |  | 0.868 |
| - Tremor subscore, median (IQR) ^f^ | 2.0 (3.0) | - | - |  | 2.0 (4.0) | - | - |  | 0.571 |
| - Rigidity subscore, median (IQR) ^f^ | 4.0 (4.0) | - | - |  | 4.0 (5.0) | - | - |  | 0.575 |
| - Bradykinesia subscore, median (IQR) ^f^ | 10.0 (8.0) | - | - |  | 10.0 (9.0) | - | - |  | 0.641 |
| - Axial subscore, median (IQR) ^f^ | 3.0 (2.0) | - | - |  | 3.0 (3.0) | - | - |  | 0.268 |
| **Non-motor symptoms** |  |  |  |  |  |  |  |  |  |
| - NMSS score, median (IQR) ^f^ | 27.5 (22.0) | - | - |  | 23.0 (27.0) | - | - |  | 0.421 |
| - MoCA score, median (IQR) ^f^ | 23.0 (7.0) | - | - |  | 22.0 (7.0) | - | - |  | 0.794 |
| - RBDSQ score, median (IQR) ^f^ | 1.0 (4.0) | - | - |  | 1.0 (3.0) | - | - |  | 0.715 |
| - SS-12 score, median (IQR) ^f^ | 5.0 (4.0) | - | - |  | 5.0 (3.0) | - | - |  | 0.846 |

Note: ^a^ *p* value: comparison between the follow-up group and the lost to follow-up group in G allele carriers (AG+GG), ^b^ *p* value: comparison between the follow-up group and the lost to follow-up group in G allele non-carriers (AA), ^c^ *p* value: comparison between the G allele carriers and G allele non-carriers included in the longitudinal analysis (AG+GG vs. AA). ^d^ Chi square was used; ^e^ Student's *t*-test was used; ^f^ Mann–Whitney U test was used.

Abbreviations: n, number; SD, standard deviation; IQR, interquartile range; LEDDs, L-Dopa equivalent daily dosages; MDS-UPDRS-III, Part III of Movement Disorders Society Unified Parkinson's Disease Rating Scale; NMSS, Non-motor Symptoms Scale; MoCA, Montreal Cognitive Assessment; RBDSQ, Rapid Eye Movement Sleep Behavior Disorder Screening Questionnaire; SS-12, 12-item Sniffin' Sticks test.

**Table S5.** Fixed effects of generalized linear mixed models testing the effect of different *HLA-DRB1* genotypes on motor symptom scores at 3-year follow-up (under the dominant model)

| **Fixed effects** | **MDS-UPDRS-III  total score** | |  | **Tremor subscore** | |  | **Rigidity**  **subscore** | |  | **Bradykinesia subscore** | |  | **Axial  subscore** | |
| --- | --- | --- | --- | --- | --- | --- | --- | --- | --- | --- | --- | --- | --- | --- |
|  | ***F*** | ***p* value** |  | ***F*** | ***p* value** |  | ***F*** | ***p* value** |  | ***F*** | ***p* value** |  | ***F*** | ***p* value** |
| **Model 1** |  |  |  |  |  |  |  |  |  |  |  |  |  |  |
| Time | 564.34 | **<0.001^***^** |  | 62.92 | **<0.001^***^** |  | 237.80 | **<0.001^***^** |  | 320.76 | **<0.001^***^** |  | 201.75 | **<0.001^***^** |
| Genotype | 3.69 | 0.055 |  | 1.18 | 0.277 |  | 1.70 | 0.192 |  | 2.88 | 0.090 |  | 4.22 | **0.040^*^** |
| Time × Genotype | 15.66 | **<0.001^***^** |  | 0.81 | 0.489 |  | 3.71 | **0.011^*^** |  | 5.96 | **<0.001^***^** |  | 14.68 | **<0.001^***^** |
| **Model 2** |  |  |  |  |  |  |  |  |  |  |  |  |  |  |
| Time | 457.45 | **<0.001^***^** |  | 57.52 | **<0.001^***^** |  | 207.70 | **<0.001^***^** |  | 272.83 | **<0.001^***^** |  | 167.75 | **<0.001^***^** |
| Gender | 2.28 | 0.131 |  | 0.18 | 0.672 |  | 6.76 | **0.009^**^** |  | <0.001 | 0.991 |  | 1.08 | 0.300 |
| Age | 14.58 | **<0.001^***^** |  | 0.06 | 0.805 |  | 4.06 | **0.044^*^** |  | 15.59 | **<0.001^***^** |  | 22.63 | **<0.001^***^** |
| Genotype | 4.59 | **0.032^*^** |  | 1.23 | 0.267 |  | 2.23 | 0.135 |  | 3.42 | 0.064 |  | 5.32 | **0.021** |
| Time × Genotype | 15.69 | **<0.001^***^** |  | 0.81 | 0.489 |  | 3.72 | **0.011^*^** |  | 5.97 | **<0.001^***^** |  | 14.62 | **<0.001^***^** |
| **Model 3** |  |  |  |  |  |  |  |  |  |  |  |  |  |  |
| Time | 143.30 | **<0.001^***^** |  | 17.64 | **<0.001^***^** |  | 79.44 | **<0.001^***^** |  | 107.33 | **<0.001^***^** |  | 48.72 | **<0.001^***^** |
| Gender | 1.83 | 0.176 |  | 0.23 | 0.634 |  | 6.29 | **0.012^*^** |  | 0.07 | 0.790 |  | 0.48 | 0.490 |
| Age | 9.17 | **0.002^**^** |  | 0.69 | 0.407 |  | 2.63 | 0.105 |  | 13.20 | <0.001*** |  | 16.42 | **<0.001^***^** |
| Onset age | 0.219 | 0.640 |  | 1.60 | 0.207 |  | 0.14 | 0.709 |  | 1.56 | 0.212 |  | 0.74 | 0.389 |
| Disease course | 27.287 | **<0.001^***^** |  | 32.82 | **<0.001^***^** |  | 8.80 | **0.003^**^** |  | 7.16 | **0.008^**^** |  | 25.73 | **<0.001^***^** |
| LEDDs | 19.513 | **<0.001^***^** |  | 0.25 | 0.615 |  | 5.57 | **0.018^*^** |  | 9.67 | **0.002^**^** |  | 39.33 | **<0.001^***^** |
| Genotype | 3.95 | **0.047^*^** |  | 1.11 | 0.292 |  | 1.79 | 0.181 |  | 2.77 | 0.096 |  | 4.13 | **0.042^*^** |
| Time × Genotype | 13.93 | **<0.001^***^** |  | 0.76 | 0.518 |  | 3.18 | **0.023^*^** |  | 5.14 | **0.002^**^** |  | 12.93 | **<0.001^***^** |

Note: Genotypes were grouped under the dominant model of *HLA-DRB1* rs660895. Model 1—unadjusted model; Model 2—minimally adjusted model, adjusted for age and gender; Model 3—fully adjusted model, adjusted for age, gender, onset age, disease course, and LEDDs. Boldface indicates statistical significance.

Abbreviations: LEDDs, L-Dopa equivalent daily dosages; MDS-UPDRS-III, Part III of Movement Disorders Society Unified Parkinson's Disease Rating Scale.

Boldface indicates statistical significance (^*^ *p* < 0.05, ^**^ *p* < 0.01, ^***^ *p* <0.001).

**Table S6.** Fixed effects of generalized linear mixed models testing the effect of different *HLA-DRB1* genotypes on non-motor symptoms scores at 3-year follow-up (under the dominant model)

|  | **NMSS score** | |  | **MoCA score** | |  | **RBD-SQ score** | |  | **SS-12 score** | |
| --- | --- | --- | --- | --- | --- | --- | --- | --- | --- | --- | --- |
| **Fixed effects** | ***F*** | ***p*** value |  | ***F*** | ***p*** value |  | ***F*** | ***p*** value |  | ***F*** | ***p*** value |
| **Model 1** |  |  |  |  |  |  |  |  |  |  |  |
| Time | 324.12 | **<0.001**^***^ |  | 378.69 | **<0.001**^***^ |  | 260.64 | **<0.001**^***^ |  | 260.64 | **<0.001**^***^ |
| Genotype | 1.08 | 0.298 |  | 1.66 | 0.198 |  | 0.02 | 0.890 |  | 0.02 | 0.890 |
| Time × Genotype | 5.59 | **0.001**^**^ |  | 5.41 | **0.001**^**^ |  | 3.17 | **0.024**^*^ |  | 3.17 | **0.024**^*^ |
| **Model 2** |  |  |  |  |  |  |  |  |  |  |  |
| Time | 256.86 | **<0.001**^***^ |  | 269.96 | **<0.001**^***^ |  | 216.06 | **<0.001**^***^ |  | 202.60 | **<0.001**^***^ |
| Gender | 0.21 | 0.647 |  | 11.70 | **0.001**^**^ |  | 2.77 | 0.096 |  | 1.27 | 0.259 |
| Age | 10.72 | **0.001**^**^ |  | 25.46 | **<0.001**^***^ |  | 7.84 | **0.005** |  | 26.03 | **<0.001**^***^ |
| Genotype | 1.00 | 0.317 |  | 2.44 | 0.119 |  | <0.001 | 0.982 |  | 2.23 | 0.136 |
| Time × Genotype | 6.37 | **<0.001**^***^ |  | 5.17 | **0.001**^**^ |  | 3.17 | **0.024** |  | 4.68 | **0.003**^*^ |
| **Model 3** |  |  |  |  |  |  |  |  |  |  |  |
| Time | 50.45 | **<0.001**^***^ |  | 76.60 | **<0.001**^***^ |  | 72.42 | **<0.001**^***^ |  | 90.58 | **<0.001**^***^ |
| Gender | 0.05 | 0.829 |  | 13.79 | **<0.001**^***^ |  | 2.50 | 0.114 |  | 1.26 | 0.261 |
| Age | 11.18 | **0.001**^**^ |  | 17.99 | **<0.001**^***^ |  | 3.77 | 0.052 |  | 9.45 | **0.002**^**^ |
| Onset age | 1.85 | 0.175 |  | 1.18 | 0.279 |  | 0.01 | 0.945 |  | 0.33 | 0.563 |
| Disease course | 29.80 | **<0.001**^***^ |  | 0.18 | 0.673 |  | 10.59 | **0.001**^**^ |  | 0.05 | 0.828 |
| LEDDs | 11.23 | **0.001**^**^ |  | 22.61 | **<0.001**^***^ |  | 11.10 | **0.001**^**^ |  | 6.72 | **0.010**^*^ |
| Genotype | 0.71 | 0.400 |  | 1.61 | 0.205 |  | 0.04 | 0.851 |  | 1.86 | 0.172 |
| Time × Genotype | 6.08 | **<0.001**^***^ |  | 3.71 | **0.011**^*^ |  | 2.69 | **0.045**^*^ |  | 4.16 | **0.006**^**^ |

Note: Genotypes were grouped under the dominant model of *HLA-DRB1* rs660895. Model 1—unadjusted model; Model 2—minimally adjusted model, adjusted for age and gender; Model 3—fully adjusted model, adjusted for age, gender, onset age, disease course, and LEDDs. Boldface indicates statistical significance.

Abbreviations: LEDDs, L-Dopa equivalent daily dosages; NMSS, Non-motor Symptoms Scale; MoCA, Montreal Cognitive Assessment; RBDSQ, Rapid Eye Movement Sleep Behavior Disorder Screening Questionnaire; SS-12, 12-item Sniffin' Sticks test.

Boldface indicates statistical significance (^*^ *p* < 0.05, ^**^ *p* < 0.01, ^***^ *p* <0.001).

**Table S7.** Review of studies in AD or PD identifying significant association at the HLA loci

| **Study** | **Population** | **Gene** | **SNP** | **Allele** | **Effect** | **OR** | ***p* value** |
| --- | --- | --- | --- | --- | --- | --- | --- |
| Hamza et al. 2010 (1) | Caucasian | *HLA-DRA* | rs3129882 | G/A | Risk-A | 1.26 | 1.9×10^−10^ |
| International Parkinson Disease Genomics Consortium. 2011 (2) | American,  European | *HLA-DRB5* | chr6:32588205 | G/A | Protective-A | 0.70 | 3×10^−8^ |
| Guo et al. 2011(3) | Chinese | *HLA-DRA* | rs3129882 | G/A | Risk-A | 1.37 | 0.016 |
| Ahmed et al. 2012 (4) | French | *HLA-DRB1* | rs660895 | A/G | Protective-G | 0.86 | <1×10^−4^ |
| William et al. 2013 (5) | European | *HLA-DRA*  *HLA-DRB1* | rs3129882  rs9268515  rs2395163  rs75855844  rs660895 | A/G  G/C  T/C  A/G  A/G | Risk-G  Protective-C  Protective-C  Protective-G  Protective-G | 1.30  0.75  0.81  0.78  0.80 | 9×10^−11^  8×10^−8^  3×10^−11^  8×10^−9^  8×10^−7^ |
| Nalls et al. 2014 (6) | European | *HLA-DQB1* | rs9275326 | C/T | Protective-T | 0.826 | 1.19×10^-12^ |
| Chuang et al. 2017 (7) | Denmark,  French | *HLA-DRB1* | rs660895 | A/G | Protective-G | AG: 0.81  GG: 0.56 | 0.003 |
| Hollenbach et al. 2019 (8) | European | *HLA-DRB1*  *HLA-DQB1* | 04:01  03:02 | /  / | /  / | 0.78  0.78 | 0.007  0.006 |
| Chang et al. 2020 (9) | Taiwan | *HLA-DRA* | rs2395163 | T/C | Protective-C  (in male) | 0.58 | 0.009 |
| Yu et al. 2021 (10) | European | *HLA-DRA*  *HLA-DRB1*  *HLA-DRB5*  *HAL-DQB1* | rs2395163  rs17425622  rs112485576  rs9275326 | T/C  T/C  G/A  C/T | Protective-C  Protective-C  Protective-A  Protective-T | 0.94  0.92  0.87  0.92 | 3.235×10^13^  1.96×10^-9^  5×10^-13^  5×10^-13^ |
| Naito et al. 2021 (11) | European,  East Asian | *HLA-DRB1* | rs9269951  rs1136759 rs1136758  (haplotypes) | C/A  C/A/G/T  T/A/C/G | Risk-ACG, CCG, CCT  Protective-ATG  Risk-AAA  Risk-ACT, AGA, CGA  Risk-ATA | / | 9.1 × 10^-5^  6.0 × 10^-15^  0.0036  0.030  0.027 |
|  |  | *HLA-B* | rs41548914  rs41546313  rs1131204  (haplotypes) | G/A/T  G/A/C  C/G/T | Risk- AGC, GGC, TGC  Protective-AGT, GGT, TGT |  | 1.0 × 10^-7^  4.8× 10^-7^ |
| Le Guen et al, 2023 (12) | European,  East Asian | *HLA-DRB1*  *HLA-DQB1*  *HLA-DQA1* | 04:01  04:02  04:03  04:04  04:07  01:01  15:01  03:02  03:01 | /  /  / | Protective  Protective  Protective  Protective  Protective  Risk  Risk  Protective  Protective | 0.92  0.92  0.89  0.84  0.79  1.05  1.06  0.91  0.89 | 2.0×10^-8^  0.02  0.01  1.5×10^-11^  7.3×10^-4^  7.0×10^-3^  5.0×10^-4^  2.6×10^-14^  2.5×10^-20^ |

## SUPPLEMENTARY REFERENCES

1. Hamza TH, Zabetian CP, Tenesa A, Laederach A, Montimurro J, Yearout D, et al. Common genetic variation in the HLA region is associated with late-onset sporadic Parkinson's disease. *Nat Genet*. 2010;42(9):781-5. doi: 10.1038/ng.642

2. International Parkinson Disease Genomics Consortium, Nalls MA, Plagnol V, Hernandez DG, Sharma M, Sheerin UM, et al. Imputation of sequence variants for identification of genetic risks for Parkinson's disease: a meta-analysis of genome-wide association studies. *Lancet*. 2011;377(9766):641-9. doi: 10.1016/s0140-6736(10)62345-8

3. Guo Y, Deng X, Zheng W, Xu H, Song Z, Liang H, et al. HLA rs3129882 variant in Chinese Han patients with late-onset sporadic Parkinson disease. *Neurosci Lett*. 2011;501(3):185-7. doi: 10.1016/j.neulet.2011.05.245

4. Ahmed I, Tamouza R, Delord M, Krishnamoorthy R, Tzourio C, Mulot C, et al. Association between Parkinson's disease and the HLA-DRB1 locus. *Mov Disord*. 2012;27(9):1104-10. doi: 10.1002/mds.25035

5. Wissemann WT, Hill-Burns EM, Zabetian CP, Factor SA, Patsopoulos N, Hoglund B, et al. Association of Parkinson disease with structural and regulatory variants in the HLA region. *Am J Hum Genet*. 2013;93(5):984-93. doi: 10.1016/j.ajhg.2013.10.009

6. Nalls MA, Pankratz N, Lill CM, Do CB, Hernandez DG, Saad M, et al. Large-scale meta-analysis of genome-wide association data identifies six new risk loci for Parkinson's disease. *Nat Genet*. 2014;46(9):989-93. doi: 10.1038/ng.3043

7. Chuang YH, Lee PC, Vlaar T, Mulot C, Loriot MA, Hansen J, et al. Pooled analysis of the HLA-DRB1 by smoking interaction in Parkinson disease. *Ann Neurol*. 2017;82(5):655-64.

8. Hollenbach JA, Norman PJ, Creary LE, Damotte V, Montero-Martin G, Caillier S, et al. A specific amino acid motif of HLA-DRB1 mediates risk and interacts with smoking history in Parkinson's disease. Proc Natl Acad Sci U S A. 2019;116(15):7419-24.

9. Chang KH, Wu YR, Chen YC, Fung HC, Chen CM. Association of genetic variants within HLA-DR region with Parkinson's disease in Taiwan. *Neurobiol Aging*. 2020;87:140.e13-.e18. doi: 10.1016/j.neurobiolaging.2019.11.002

10. Yu E, Ambati A, Andersen MS, Krohn L, Estiar MA, Saini P, et al. Fine mapping of the HLA locus in Parkinson's disease in Europeans. NPJ Parkinsons Dis. 2021;7(1):84. doi: 10.1038/s41531-021-00231-5

11. Naito T, Satake W, Ogawa K, Suzuki K, Hirata J, Foo JN, et al. Trans-Ethnic Fine-Mapping of the Major Histocompatibility Complex Region Linked to Parkinson's Disease. *Mov Disord*. 2021;36(8):1805-14. doi: 10.1002/mds.28583

12. Le Guen Y, Luo G, Ambati A, Damotte V, Jansen I, Yu E, et al. Multiancestry analysis of the HLA locus in Alzheimer's and Parkinson's diseases uncovers a shared adaptive immune response mediated by HLA-DRB1*04 subtypes. *Proc Natl Acad Sci U S A*. 2023;120(36):e2302720120. doi: 10.1073/pnas.2302720120
